# Models of cell signaling uncover molecular mechanisms of high-risk neuroblastoma and predict disease outcome

**DOI:** 10.1186/s13062-018-0219-4

**Published:** 2018-08-22

**Authors:** Marta R. Hidalgo, Alicia Amadoz, Cankut Çubuk, José Carbonell-Caballero, Joaquín Dopazo

**Affiliations:** 1 Clinical Bioinformatics Area, Fundación Progreso y Salud (FPS), CDCA, Hospital Virgen del Rocio, c/Manuel Siurot s/n, 41013 Sevilla, Spain; 2 Igenomix S.A. Ronda Narciso Monturiol, 11 B, Parque Tecnológico Paterna, 46980 Paterna, Valencia Spain; 3 Centre for Genomic Regulation, Carrer del Dr. Aiguader, 88, 08003 Barcelona, Spain; 4 Functional Genomics Node (INB). FPS, Hospital Virgen del Rocio, c/Manuel Siurot s/n, 41013 Sevilla, Spain; 5 Bioinformatics in Rare Diseases (BiER), Centro de Investigación Biomédica en Red de Enfermedades Raras (CIBERER), FPS, Hospital Virgen del Rocio, c/Manuel Siurot s/n, 41013 Sevilla, Spain

## Abstract

**Background:**

Despite the progress in neuroblastoma therapies the mortality of high-risk patients is still high (40–50%) and the molecular basis of the disease remains poorly known. Recently, a mathematical model was used to demonstrate that the network regulating stress signaling by the c-Jun N-terminal kinase pathway played a crucial role in survival of patients with neuroblastoma irrespective of their MYCN amplification status. This demonstrates the enormous potential of computational models of biological modules for the discovery of underlying molecular mechanisms of diseases.

**Results:**

Since signaling is known to be highly relevant in cancer, we have used a computational model of the whole cell signaling network to understand the molecular determinants of bad prognostic in neuroblastoma. Our model produced a comprehensive view of the molecular mechanisms of neuroblastoma tumorigenesis and progression.

**Conclusion:**

We have also shown how the activity of signaling circuits can be considered a reliable model-based prognostic biomarker.

**Reviewers:**

This article was reviewed by Tim Beissbarth, Wenzhong Xiao and Joanna Polanska. For the full reviews, please go to the Reviewers’ comments section.

**Electronic supplementary material:**

The online version of this article (10.1186/s13062-018-0219-4) contains supplementary material, which is available to authorized users.

## Background

Neuroblastoma is a tumor derived from primitive cells of the sympathetic nervous system that, despite advances in its treatment still has a poor survival for high-risk patients [1]. Risk groups are defined according to disease stage, patient age, and *MYCN* amplification status [2]. Although the use of biomarkers has demonstrated clinical utility, they represent statistical associations to clinical parameters and frequently lack any mechanistic relationship with the molecular mechanisms responsible for tumorigenesis or therapeutic response. On the contrary, signaling pathways control cell behavior and constitute the mechanisms that ultimately determine the fate of cancer cells. In fact, in a recent study, a mathematical model of the JNK signaling dynamics has demonstrated that this pathway plays a major role in neuroblastoma [3]. Moreover, the study demonstrated that the activity of the JNK signaling pathway showed a more significant correlation with patient survival than those shown by any of their constituent genes. Therefore, these results revealed how JNK signaling dynamics represents an innovative type of model-based biomarker that efficiently predicts neuroblastoma patient prognostic across different individual molecular backgrounds defined by conventional single gene biomarkers. This concept has been recently extended to other cancers where computational models demonstrated that the activity of specific circuits of signaling pathways related to diverse cancer hallmarks [4] provided a robust prediction of patient survival [5]. Moreover, the accuracy of the prediction obtained using the activity of the signaling circuit surpassed the conventional predictions based solely on the activities of their constituent proteins, clearly demonstrating that not only the levels of signaling individual nodes but also the network topology of the signaling circuit and thus the nonlinear properties of a signal response should ideally be captured in a biomarker in order to produce a robust prediction of patient outcome [5]. Furthermore, this type of models have proven to be superior to other pathway-based models [6].

Here, we have used generalized computational models covering all the signaling activity related with cancer hallmarks and other cancer-related signaling pathways. Such computational models use gene expression data to produce a realistic estimation of signaling circuit activity within pathways [5], which can subsequently be used to discover the molecular mechanisms behind the differences between patients with and without *MYCN* amplification as well as to uncover the determinants of survival in neuroblastoma patients.

## Results

### Data processing

A gene expression matrix with expression values quantified as log_2_(1 + FPKM) were downloaded from the GEO database. In order to correct batch effect the COMBAT [7] method was used. The expression values were further normalized between 0 and 1 to run the software implementing the models.

### Molecular mechanisms behind the MYCN amplification biomarker

**Fig. 1:**
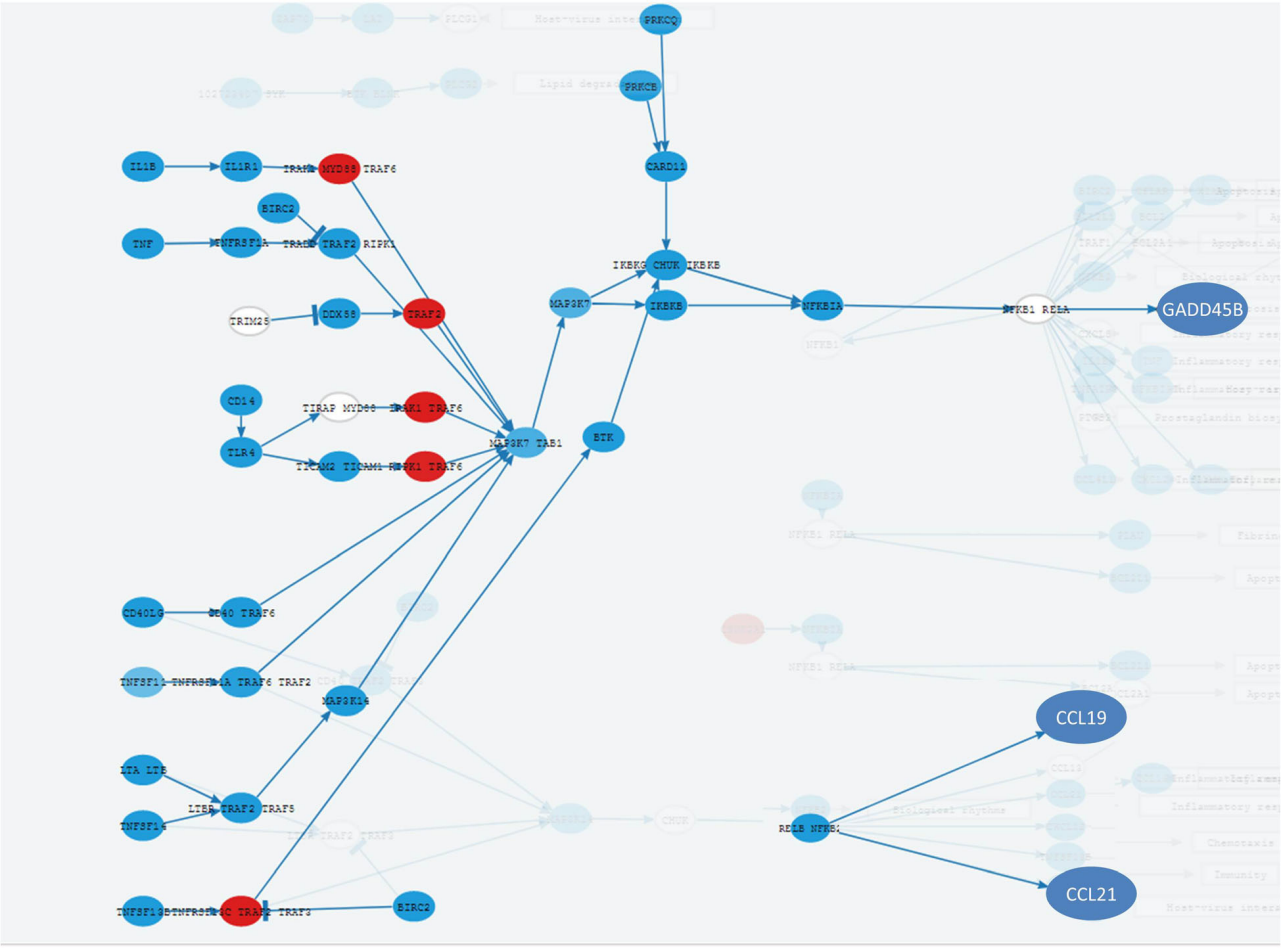
Three signaling circuits ending in the proteins CCL19, CCL21 and GADD45B highlighted within the whole NF-kappa B signaling pathway. The circuits are significantly deactivated in patients with MYCN amplification when compared to patients without such biomarker. The results and the representation have been obtained with the program HiPathia [5]. Blue and red nodes indicate genes downregulated and upregulated, respectively. Blue arrows depict the circuits in which signal transduction is inhibited

Since *MYCN* amplification is a known biomarker of bad prognostic [2] we were interested in understanding the molecular basis of such pathological phenotype. To achieve so, we carried out a differential signaling activity test comparing patients with *MYCN* amplification to those ones lacking this biomarker. Overall, our results document extensive differences at the level of signaling activity between patients with different *MYCN* amplification status. Specifically, patients with *MYCN* amplification seem to inhibit the *JNK* pathway, necessary for cell apoptosis, confirming in this way previous observations [3]. The mechanism for JNK inhibition seems complex and involves the participation of several important pathways such as Ras pathway, Apoptosis, MAPK signaling pathway and NF-kappa B signaling pathways, among others (see Table 1). In particular, the NF-kappa B signaling pathway significantly deactivates three signalling circuits ending in the proteins *CCL19*, *CCL21* and *GADD45B,* as depicted in Fig. 1. Also, the MAPK signaling pathway, together with the circuits that transduce signal to *MAPK8* within *Ras*, *Fc epsilon RI* and *cAMP* signaling pathways, seems to play an important role as mechanisms for the inactivation of the JNK pathway.

**Table 1:** Circuits that deactivate the *JNK* cascade in patients with *MYCN* amplification

Circuit (Pathway and effector protein)	Status	FDR *p*-value	GO ID	GO Definition
Ras signaling pathway: MAPK8	DOWN	4.31E-29	GO:0007254	JNK cascade
Fc epsilon RI signaling pathway: MAPK8	DOWN	1.23E-15	GO:0007254	JNK cascade
cAMP signaling pathway: MAPK8	DOWN	4.72E-08	GO:0007254	JNK cascade
Apoptosis: GADD45G	DOWN	1.07E-29	GO:0046330	positive regulation of JNK cascade
MAPK signaling pathway: MAP 4 K2	DOWN	1.68E-24	GO:0046330	positive regulation of JNK cascade
NF-kappa B signaling pathway: CCL19	DOWN	2.36E-22	GO:0046330	positive regulation of JNK cascade
NF-kappa B signaling pathway: GADD45B	DOWN	3.83E-21	GO:0046330	positive regulation of JNK cascade
NF-kappa B signaling pathway: CCL21	DOWN	8.43E-16	GO:0046330	positive regulation of JNK cascade
p53 signaling pathway: GADD45G	UP	1.70E-07	GO:0046330	positive regulation of JNK cascade

**Fig. 2:**
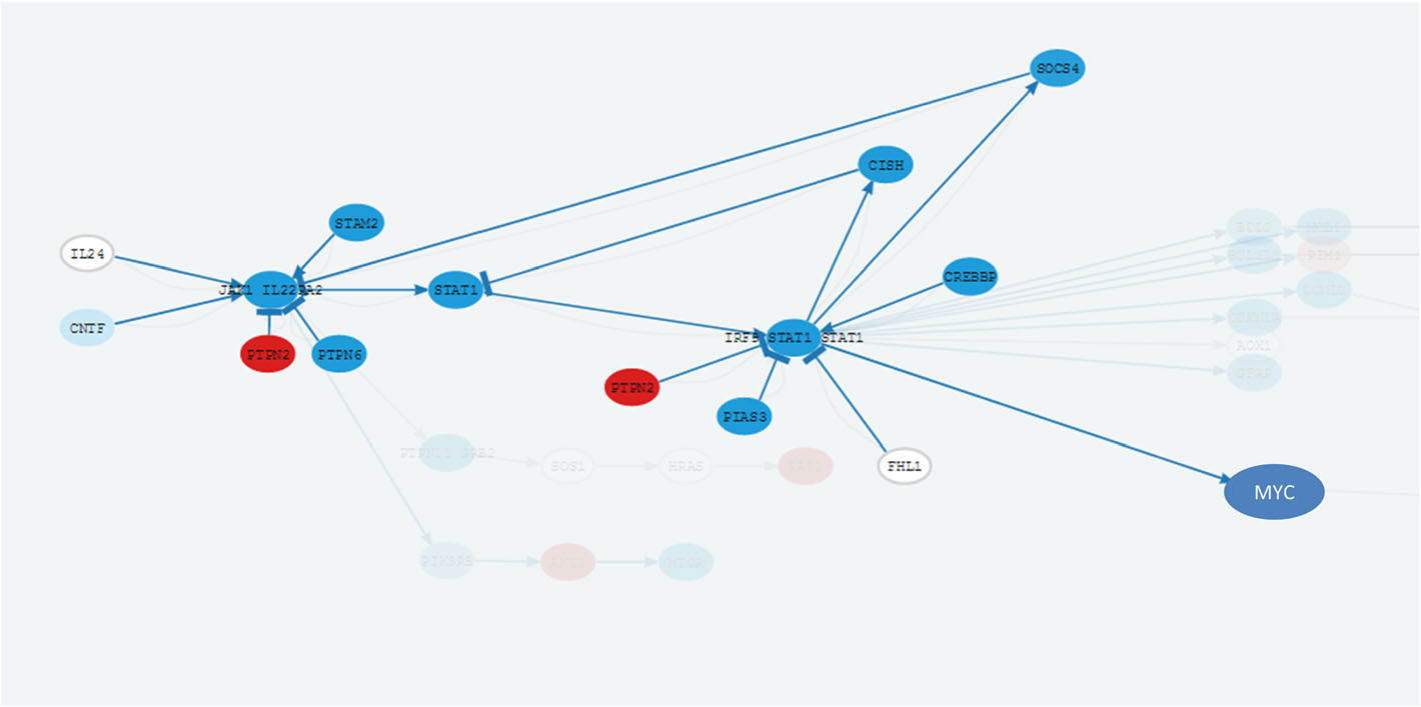
JACK-STAT signaling pathway with the circuit ending in *MYC* protein. That triggers response to DNA damage. Significantly (FDR-adj. *p*-value = 1.94 × 10^− 32^) deactivated in patients with *MYCN* amplification. The results and the representation have been obtained with the program HiPathia [5]. Blue and red nodes indicate genes downregulated and upregulated, respectively, in patients with *MYCN* amplification. The deactivations of nodes that transmit the signal concomitantly with the activation of signal repressor genes strongly suggest the actuation of a regulatory program to inhibit the signal

Another well-defined mechanism characteristic of patients with *MYCN* amplification seems to be defective DNA repair. Again, the mechanism seems complex and mediated by many different pathways, which is not surprising, given that DNA repair must be a robust mechanism. A total of 5 circuits belonging to the pathways Jak-STAT, MAPK, ErbB, Wnt and Hippo signaling pathways present a highly significant deactivation in patients with *MYCN* amplification (see Table 2). As an example, Fig. 2 shows the inhibition in the JACK-STAT pathway. Remarkably, the effector of all these circuits is the *MYC* protein, which seems to be the counterpart of *MYCN* in patients with *MYCN*-nonamplified neuroblastomas. In fact, *BMI1* expression, a gene, whose suppression resulted in significantly greater inhibition of cell growth, correlated with *MYCN* levels in *MYCN*-amplified neuroblastoma cells, and with *MYC* levels in the *MYCN*-nonamplified group [8].

**Table 2:** Circuits that deactivate DNA repair and related cell functions

Circuit (Pathway and effector protein)	Status	FDR p-value	GO ID	GO Definition
Jak-STAT signaling pathway: MYC	DOWN	1.94E-32	GO:2001022	positive regulation of response to DNA damage stimulus
MAPK signaling pathway: MYC	DOWN	1.39E-26	GO:2001022	positive regulation of response to DNA damage stimulus
ErbB signaling pathway: MYC	DOWN	7.15E-24	GO:2001022	positive regulation of response to DNA damage stimulus
Wnt signaling pathway: MYC	DOWN	7.78E-22	GO:2001022	positive regulation of response to DNA damage stimulus
Hippo signaling pathway: MYC	DOWN	7.12E-13	GO:2001022	positive regulation of response to DNA damage stimulus
Jak-STAT signaling pathway: MYC	DOWN	1.94E-32	GO:0006338	chromatin remodeling
MAPK signaling pathway: MYC	DOWN	1.39E-26	GO:0006338	chromatin remodeling
ErbB signaling pathway: MYC	DOWN	7.15E-24	GO:0006338	chromatin remodeling
Wnt signaling pathway: MYC	DOWN	7.78E-22	GO:0006338	chromatin remodeling
Hippo signaling pathway: MYC	DOWN	7.12E-13	GO:0006338	chromatin remodeling

The rest of processes that can be considered as cancer hallmarks [4] have an inconclusive distribution between the two groups of neuroblastomas. For example, angiogenesis seem to be activated in MYCN-amplified patients through circuits in Apoptosis, cGMP-PKG and PI3K-Akt signaling pathways but other circuits in other pathways (HIF-1, NF-kappa B and P53) seem to deactivate it (see Table 3).

**Table 3:** Circuits with different effects on angiogenesis

Circuit (Pathway and effector protein)	Status	FDR p-value	GO ID	GO Definition
Apoptosis: FASLG	DOWN	1.44E-22	GO:0016525	negative regulation of angiogenesis
PI3K-Akt signaling pathway: FASLG	DOWN	1.18E-21	GO:0016525	negative regulation of angiogenesis
p53 signaling pathway: THBS1	UP	4.43E-09	GO:0016525	negative regulation of angiogenesis
cGMP-PKG signaling pathway: GTF2I	DOWN	3.40E-06	GO:0016525	negative regulation of angiogenesis
HIF-1 signaling pathway: TEK	DOWN	8.85E-27	GO:0045766	positive regulation of angiogenesis
HIF-1 signaling pathway: VEGFA	DOWN	2.20E-25	GO:0045766	positive regulation of angiogenesis
NF-kappa B signaling pathway: PLCG1	DOWN	3.62E-17	GO:0045766	positive regulation of angiogenesis

These results documents that while patients with *MYCN* amplification have characteristic signaling activities that trigger processes which contribute to bad prognostic, such as the inhibition of the JNK pathway or potentially defective DNA repair, much of the cancer hallmarks are not exclusive of this group. Therefore we investigate what are the mechanisms behind patient mortality irrespective of *MYCN* amplification status in the following section.

### Molecular mechanisms that determine patient survival

For each circuit, patients irrespective of its *MYCN* amplification status were divided into two groups: 10% highest circuit activity patients and the rest and K-M curves were plotted and tests were applied to detect significant differences in survival. The same procedure was repeated with the 10% lowest circuit activity patients (see Methods).

**Fig. 3:**
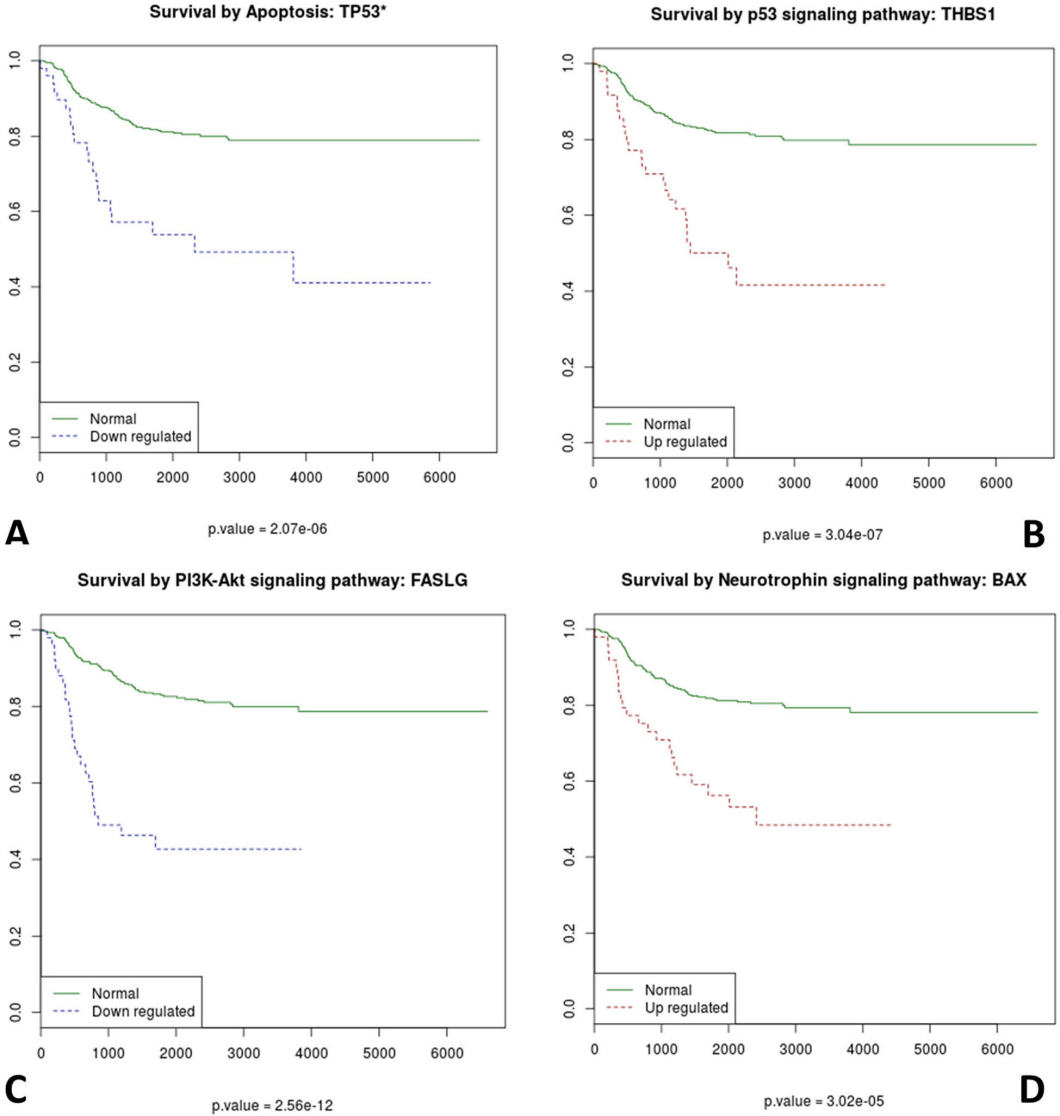
K-M plots of patients with **a**) inhibition of apoptosis via inhibition of a circuit of the Apoptosis pathway ending in the *TP53* gene; **b**) activation of metastatic activity by activation of a circuit of the p53 signaling pathway ending in the *THBS1* gene; **c**) activation of angiogenesis via the inhibition *FASLG* through the corresponding circuit in the PI3K-Atk signaling pathway; **d**) apparent inhibition of the immune response by specific apoptosis induction of B cells via the circuit in the Neutrophin pathway that activates the known apoptotic protein BAX

We were able to detect numerous processes activated and deactivated with a strong significant association to survival that could easily be associated to known cancer hallmarks (Table 4). Inhibition of apoptosis is a recognized cancer hallmark, whose mechanism of deactivation is disclosed here. Negative regulation of apoptosis is induced in patients with activated signaling circuits in the PI3K-Akt signaling pathway (PI3K-Akt signaling pathway: *BCL2L1*). Apoptosis is massively inhibited through the inhibition of several circuits in the following pathways: Apoptosis (see Fig. 3a for an example), ErbB, Hippo, Jak-STAT, MAPK, mTOR, NF-kappa B, NOD-like receptor, PI3K-Akt, Ras, T cell receptor, Tight junction, Toll-like receptor and Wnt (Table 4). Interestingly, 5 circuits belonging to pathways Apoptosis, Fc epsilon RI, NF-kappa B, MAPK and Ras (see Table 4) are inhibiting apoptosis via JNK inhibition, which provide a mechanism for this observation [3]. Patients with the corresponding activations or deactivations of these circuits that ultimately deactivate apoptosis have a significantly higher mortality (see Table 4).

**Table 4:** Circuits significantly associated to bad prognostic

Circuit (Pathway and effector protein)	Status	FDR p-val	GO ID	GO definition	Cancer hallmark
PI3K-Akt signaling pathway: BCL2L1	UP	5.13E-07	GO:1900118	negative regulation of execution phase of apoptosis	Apoptosis inhibition
Axon guidance: PAK4	UP	1.47E-05	GO:2001271	negative regulation of cysteine-type endopeptidase activity involved in execution phase of apoptosis	Apoptosis inhibition
PI3K-Akt signaling pathway: BCL2L1	UP	0.000159	GO:1900118	negative regulation of execution phase of apoptosis	Apoptosis inhibition
Adherens junction: LEF1 CTNNB1	DOWN	7.35E-07	GO:0043065	positive regulation of apoptotic process	Apoptosis inhibition
Apoptosis: BCL2L11	DOWN	2.28E-11	GO:2000271	positive regulation of fibroblast apoptotic process	Apoptosis inhibition
Apoptosis: FAS	DOWN	3.43E-06	GO:0043065	positive regulation of apoptotic process	Apoptosis inhibition
Apoptosis: FASLG	DOWN	9.88E-11	GO:2000353	positive regulation of endothelial cell apoptotic process	Apoptosis inhibition
Apoptosis: GADD45G	DOWN	5.11E-11	GO:0043065	positive regulation of apoptotic process	Apoptosis inhibition
Apoptosis: TP53	DOWN	2.07E-06	GO:0090200	positive regulation of release of cytochrome c from mitochondria	Apoptosis inhibition
Axon guidance: NCK1 PAK4	DOWN	0.0003919	GO:1902237	positive regulation of endoplasmic reticulum stress-induced intrinsic apoptotic signaling pathway	Apoptosis inhibition
ErbB signaling pathway: MYC	DOWN	6.05E-07	GO:0043280	positive regulation of cysteine-type endopeptidase activity involved in apoptotic process	Apoptosis inhibition
Hippo signaling pathway: MYC	DOWN	0.0006402	GO:0043280	positive regulation of cysteine-type endopeptidase activity involved in apoptotic process	Apoptosis inhibition
Jak-STAT signaling pathway: MYC	DOWN	2.38E-14	GO:0043280	positive regulation of cysteine-type endopeptidase activity involved in apoptotic process	Apoptosis inhibition
MAPK signaling pathway: MYC	DOWN	1.46E-10	GO:0043280	positive regulation of cysteine-type endopeptidase activity involved in apoptotic process	Apoptosis inhibition
MAPK signaling pathway: STK3	DOWN	2.47E-07	GO:0043065	positive regulation of apoptotic process	Apoptosis inhibition
mTOR signaling pathway: RPS6	DOWN	2.77E-11	GO:0043065	positive regulation of apoptotic process	Apoptosis inhibition
NF-kappa B signaling pathway: GADD45B	DOWN	5.23E-07	GO:0043065	positive regulation of apoptotic process	Apoptosis inhibition
NOD-like receptor signaling pathway: PYCARD CASP1	DOWN	7.86E-05	GO:0043280	positive regulation of cysteine-type endopeptidase activity involved in apoptotic process	Apoptosis inhibition
PI3K-Akt signaling pathway: BCL2L11	DOWN	0.0003220	GO:2000271	positive regulation of fibroblast apoptotic process	Apoptosis inhibition
PI3K-Akt signaling pathway: FASLG	DOWN	2.56E-12	GO:2000353	positive regulation of endothelial cell apoptotic process	Apoptosis inhibition
PI3K-Akt signaling pathway: RPS6	DOWN	3.43E-06	GO:0043065	positive regulation of apoptotic process	Apoptosis inhibition
Ras signaling pathway: STK4 STK4	DOWN	0.0001864	GO:0043065	positive regulation of apoptotic process	Apoptosis inhibition
T cell receptor signaling pathway: CD40LG	DOWN	0.0002348	GO:2000353	positive regulation of endothelial cell apoptotic process	Apoptosis inhibition
Tight junction: TJP1 CTNNB1 CTNNA1	DOWN	0.0004428	GO:0043065	positive regulation of apoptotic process	Apoptosis inhibition
Toll-like receptor signaling pathway: CCL5	DOWN	1.89E-08	GO:0070234	positive regulation of T cell apoptotic process	Apoptosis inhibition
Wnt signaling pathway: MYC	DOWN	5.12E-05	GO:0043280	positive regulation of cysteine-type endopeptidase activity involved in apoptotic process	Apoptosis inhibition
					Apoptosis inhibition
Apoptosis: GADD45G	DOWN	5.11E-11	GO:0046330	positive regulation of JNK cascade	Apoptosis inhibition via JNK
Fc epsilon RI signaling pathway: MAPK8	DOWN	1.78E-07	GO:0007254	JNK cascade	Apoptosis inhibition via JNK
NF-kappa B signaling pathway: GADD45B	DOWN	5.23E-07	GO:0046330	positive regulation of JNK cascade	Apoptosis inhibition via JNK
MAPK signaling pathway: MAP 4 K2	DOWN	9.26E-05	GO:0046330	positive regulation of JNK cascade	Apoptosis inhibition via JNK
NF-kappa B signaling pathway: CCL21	DOWN	0.0001040	GO:0046330	positive regulation of JNK cascade	Apoptosis inhibition via JNK
Ras signaling pathway: MAPK8	DOWN	0.0003003	GO:0007254	JNK cascade	Apoptosis inhibition via JNK
Neurotrophin signaling pathway: BAX	UP	3.02E-05	GO:1990117	B cell receptor apoptotic signaling pathway	Reduced immune response
Neurotrophin signaling pathway: BAX	UP	3.02E-05	GO:0001783	B cell apoptotic process	Reduced immune response
p53 signaling pathway: THBS1	UP	3.04E-07	GO:0030335	positive regulation of cell migration	Metastasis
Wnt signaling pathway: NFATC1	UP	1.99E-06	GO:0016477	cell migration	Metastasis
PI3K-Akt signaling pathway: TP53	UP	1.17E-06	GO:0045944	positive regulation of transcription from RNA polymerase II promoter	Proliferation
Ras signaling pathway: ELK1	UP	1.38E-06	GO:0045944	positive regulation of transcription from RNA polymerase II promoter	Proliferation
Wnt signaling pathway: NFATC1	UP	1.99E-06	GO:0045944	positive regulation of transcription from RNA polymerase II promoter	Proliferation
AMPK signaling pathway: EIF4EBP1	UP	0.0001405	GO:0045947	negative regulation of translational initiation	Proliferation
ErbB signaling pathway: ELK1	UP	0.0004442	GO:0045944	positive regulation of transcription from RNA polymerase II promoter	Proliferation
Apoptosis: FASLG	DOWN	9.88E-11	GO:0000122	negative regulation of transcription from RNA polymerase II promoter	Proliferation
PI3K-Akt signaling pathway: FASLG	DOWN	2.56E-12	GO:0016525	negative regulation of angiogenesis	Angiogenesis
HIF-1 signaling pathway: ANGPT1	DOWN	1.41E-11	GO:0016525	negative regulation of angiogenesis	Angiogenesis
Apoptosis: FASLG	DOWN	9.88E-11	GO:0016525	negative regulation of angiogenesis	Angiogenesis

The patients with activation in circuit of the p53 signaling pathway ending in the *THBS1* protein, related with metastasis in gastric cancers [9], show a significantly higher mortality (FDR-adj. p-val = 3.03 × 10^− 7^) prognostic (see Fig. 3b). The prognostic is similar for patients with high activity of the circuit of the Wnt signaling pathway ending in the transcription factor *NFATc1* (FDR-adj. p-val = 1.99 × 10^− 6^), also related to tumorigenesis [10]. Both circuits seem to trigger metastasis-related cell responses.

There are three circuits that activate angiogenesis via the inhibition of the pro-apoptotic factor Fas ligand (that is inversely correlated with angiogenesis) [11] and the angiogenesis modulator *ANGPT1* [12] which appear downregulated, and consequently promoting angiogenesis, in patients with significantly high mortality (see Table 4). An example is the inhibition *FASLG* via the corresponding circuit in the PI3K-Atk signaling pathway (see Fig. 3c).

Interestingly, we found specific apoptosis induction of B cells mediated by the known apoptotic protein *BAX* [13] through the Neurotrophin signaling pathway. The activation of this circuit, which seems to be a strategy to evade immune response, is significantly associated to higher mortality in patients (FDR-adj. p-val = 3.02 × 10^− 5^; see Fig. 3d).

**Fig. 4:**
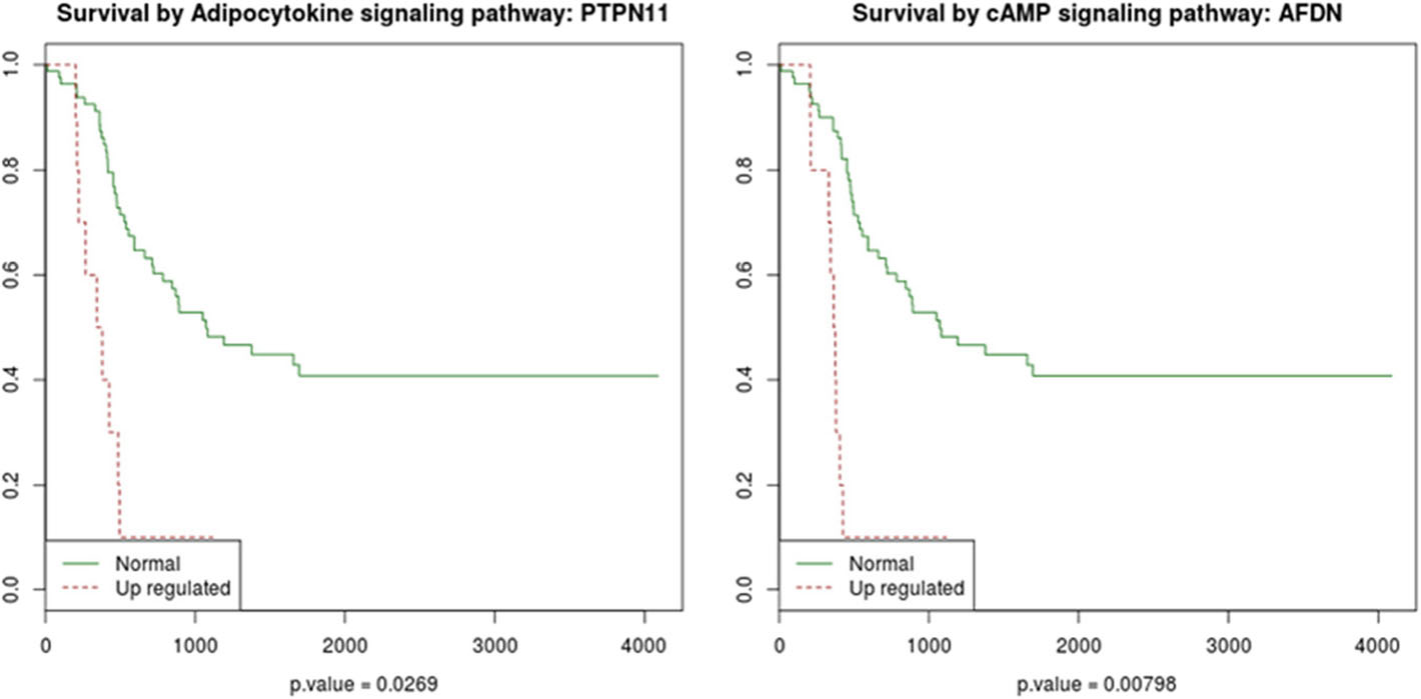
K-M plots of survival of patients with *MYCN* amplification which have downregulated Adipocytokine: *PTPN11* (left) and cAMP: *AFDN* (right) signaling circuits

We also tried to find the molecular drivers of bad prognostic specific of patients with *MYCN* amplifications. Only two circuits, *Adipocytokine: PTPN11* and *cAMP: AFDN* are significantly associated to bad prognostic (FDR-adj. *p*-values of 0.027 and 0.008, respectively; see Fig. 4). One of the effector proteins, *PTPN11* has been implicated in mitogenic activation, metabolic control, transcription regulation, and cell migration [14]. The other effector protein, *AFDN*, is the fusion partner of acute lymphoblastic leukemia (*ALL-1*) gen involved in acute myeloid leukemias with t(6;11)(q27;q23) translocation, with a known role in cell adhesion [15].

## Conclusions

It has recently been demonstrated that model-based biomarker based on the activity of the JNK pathway robustly stratified neuroblastoma patients across different molecular backgrounds [3]. Computational models have already been used to provide an understanding of the dynamics of one or a few specific signaling pathways [16–18], however, the availability of comprehensive pathway-wide models [5] that transform decontextualized transcriptomics gene expression data into signaling activities, which in turn trigger cell functions that can be linked to cancer hallmarks, provide a quantitative framework to identify neuroblastoma functional drivers. Thus, we were not only able to reproduce the results of previous modeling studies that linked the inability of activating the JNK pathway to neuroblastoma bad prognostic but also to discover the pathways upstream responsible of its inhibition. Moreover, we were able to find the involvement of numerous pathways in the activation or deactivation of numerous cell functionalities responsible of proliferation, angiogenesis, metastasis and apoptosis inhibition, four well-known cancer hallmarks. Interestingly, some of these functionalities are coordinately triggered in a way that results in a neoplastic phenotype. Although further research needs to be done to elucidate what the ultimate regulatory drivers behind such functional changes are, the widespread deregulation observed in cancer [19] acting over the wiring constrictions of the human signaling pathways must play an important role.

The use of models that quantify cell behavioral outcomes provides a unique opportunity to understand the molecular mechanisms of cancer development and progression [20], and ultimately pave the way to suggest highly specific, individualized therapeutic interventions [21, 22].

## Methods

### Data source and data preprocessing

The matrix GSE49711_SEQC_NB_TUC_G_log2.txt, with gene expression levels estimated by Cufflinks [23] and quantified as log_2_(1 + FPKM), was downloaded from the GEO database. Batch effect was corrected with COMBAT [7]. Finally, the values were normalized between 0 and 1.

### Signaling circuit activity model

Circuit activities are modelled from gene expression values as described in [5]. Briefly, KEGG pathways [24] are used to define circuits connecting receptor proteins to effector proteins. Specifically, we are using effector circuits that connect effector proteins to all the receptor proteins that can transduce the signal to them (see Additional file 1). A total of 98 KEGG pathways involving a total of 3057 genes that compose 4726 nodes were used to define a total of 1287 signaling circuits. Normalized gene expression values are used as proxies of protein activity [25–27]. The signal transmission is estimated by starting with an initial signal of 1, which is propagated along the nodes of the signaling circuits according to the following recursive rule:

1 $$ {S}_n={\upsilon}_n\bullet \left(1-\prod \limits_{s_a\in A}\left(1-{s}_a\right)\right)\cdotp \prod \limits_{s_i\in I}\left(1-{s}_i\right) $$

Where *S*_*n*_ is the signal intensity for the current node *n*, *v*_*n*_ is its normalized gene expression value, *A* is the set of activation signals (*s*_*a*_), arriving to the current node from activation edges, *I* is the set of inhibitory signals (*s*_*i*_) arriving to the node from inhibition edges [5]. In addition to circuit activities, the signal received by specific cell functions (according to either Gene Ontology [28] or Uniprot [29] definitions), triggered by more than one circuit, can also be estimated (See Additional file 2). This approach has proven to be superior to other types of pathway-based models [6].

### Statistical significance of circuit activities

Similarly to normalized gene expression values, circuit activities are measurements that do not make sense alone but rather in the context of a comparison. Thus, circuit activities can be used to compare conditions in the same way than gene expression values are used in a differential gene expression test. A Wilcoxon test is applied to assess the significance of the observed differences in circuit activities when two conditions are compared (e.g. *MYCN* amplification status). In order to correct for multiple testing effects, the False Discovery Rate (FDR) method [30] is used for the adjustment of *p*-values.

### Software implementation

The model has been implemented in a web server freely available at: http://hipathia.babelomics.org/.

Additionally, an R/Bioconductor script implementing the method is available at http://bioconductor.org/packages/devel/bioc/html/hipathia.html.

### Survival analysis

Kaplan-Meier (K-M) curves [31] are used to relate module activity to patient survival in the different cancers. The value of the activity estimated for each module in each individual was used to assess its relationship with individual patient survival. Specifically, the 10% patients with higher (or lower) circuit activities are compared to the rest of individuals to test whether high (low) circuit activity is significantly associated to survival. Calculations were carried out using the function *survdiff* from the *survival* R package (https://cran.r-project.org/web/packages/survival/). This method provides a *X*^2^ statistic [32] that is used to calculate a *p*-value. Similarly to the case of two class comparison, multiple testing effects are corrected by FDR [30].

## Reviewers’ comments

### Reviewer’s report 1

Tim Beissbarth.

### Reviewer comments

The manuscript describes an analysis on neuroblastoma data linking analysis of different pathways to molecular mechanisms in cancer and patient survival. Overall, this is an interesting and hypothesis driven modeling approach, that can better help to describe the functions of the cancer cell and thus leading to good survival models with a biological interpretation. However, I believe it also has some chances of over-fitting. I did not understand from the manuscript exactly how significance of their findings was assessed?

Author’s response: The method recodes gene expression data into circuit (sub-pathway) activities. Then, differential activities between the conditions compared can be calculated. Significance is estimated in the same way differential gene expression significance is assessed. Here we use a Wilcoxon test. We have added a subsection to the methods section.

Some external validation on an independent data-set would be of help.

Author’s response: The original HiPathia paper (Hidalgo et al., Oncotarget, 2017) contains several independent data validations.

Also comparison with other methods, either classical machine learning approaches or other pathway-structure oriented or classical gene set enrichment approaches might be interesting.

Author’s response: Actually, we have recently published a benchmarking paper in which we demonstrate that Hipathia outperforms all competing methods (Amadoz et al., 2018, Briefings in Bioinformatics, In press). We have included a sentence at the end of the first paragraph in the Background section quoting this reference in the text.

Overall, I believe this is an interesting study and modeling approach and does have some merit. Of course, to be clinically relevant more validation and further studies would be needed.

Author’s response: We cannot agree more, but obtaining clinically relevant results is outside the scope of this manuscript, that deals with the analysis of the Neuroblastoma CAMDA dataset and focuses on the throwing light on the molecular mechanisms of neuroblastoma.

If possible: - more detailed description of methods and statistical evaluation of significance - external validation on an independent data-set - comparison with other methods Critical points could also be discussed in the conclusion (to avoid overinterpretation or results).

Author’s response: As mentioned above, we have added a new subsection to the Methods section to add more detail on the statistical validation of the values obtained. Comparison with other methods has been addressed in a separated paper and the result is that HiPathia outperforms the rest of pathway-based methods.

### Reviewer’s report 2

Wenzhong Xiao

### Reviewer comments

In this manuscript, Hidalgo etc. described their work using modeling to study cell signaling mechanisms of high-risk neuroblastoma and to predict disease outcomes. The paper is well written. Using Hipathia, an approach developed by the authors previously, they extracted comprehensively 1287 signaling circuits from 98 KEGG pathways and studied their activity in the neuroblastoma data. They first examined the impact of MYCN amplification on signaling pathways in neuroblastoma and it was comforting to see that the algorithm was able to identify well defined, reasonable signaling pathways affected by the MYCN amplification.

In particular, the authors identified a set of circuits in patients with MYCN amplification that inhibit the JNK cascade. They then systematically studied each of the signaling circuits and successfully identified those which activities were significant associated with patient outcomes. The study demonstrated the feasibility of using modeling of signaling pathway activity in studying disease mechanism and developing prognostic biomarkers.

Recommendations: 1. Page 3, line 54–55. Signal from RNA-seq data has a much wider distribution than that from array data, and usually a few genes have much higher expression than the rest. Can the authors clarify how the expression values were normalized between 0 and 1? In particular, according to eq. 1 on page 7, would the few highest expression genes skew the Vn toward lower value for most of the genes?

Author’s response: As we specified in methods we downloaded from the GEO database a matrix with gene expression levels normalized by FPKM and transformed as log2(1 + FPKM) values. FPKM is a well-known and accepted normalization method for RNA-seq that accounts for sequencing depth and gene length. Finally, we re-scale the values between 0 and 1 because of HiPathia method requirements. In principle we did not observed biases due to lowly expressed genes in the gene expression values are properly normalized. Moreover, as commented, a benchmarking carried out by us pointed to HiPathia as the best performer of all the pathway based analysis methods.

Minor issues:

1. The figures, for some reason, appeared to have very low resolution. For example, in Fig. 1, the reviewer was not able to identify the proteins CCL19, CCL21 and GADD45B, nor the deactivation of these signaling circuits by NF-kappa B signaling as mentioned in text.

Author’s response: Fig. 1 depicts only the deactivated circuits within the NF-kappa B signaling pathway. We have reformulated the text and the figure because it was a bit confusing before. We have clearly labeled the genes.

2. Page 4, line 34, and other places in the text. Jack-STAT should be JAK-STAT.

Author’s response: fixed.

### Reviewer’s report 3

Joanna Polanska.

### Reviewer comments

The manuscript is devoted to study the activities of gene signaling pathways as triggers of neoplastic processes in neuroblastoma. The authors use their own computational algorithm, CCAA, previously published as [5], which enables assigning to KEGG signaling pathways a value, which describes its up or down regulation status. Activity states of gene signaling pathways are estimated on the basis of gene expression values obtained from the GEO data portal. The authors are able to demonstrate remarkable results, presented in Fig. 3, showing highly statistically significant differences between survivals of patients related to A) the status of inhibition of apoptosis via inhibition of a circuit of the Apoptosis pathway ending in the TP53 gene, B) the mechanism of activation of metastatic activity by activation of a circuit of the p53 signaling pathway ending in the THBS1 gene, C) the mechanism of activation of angiogenesis via the inhibition FASLG through the corresponding circuit in the PI3K-Atk signaling pathway, D) the mechanism of inhibition of apoptosis of B cells in the Neutrophin pathway that activates protein BAX. These mechanism are highly specific and extend the existing knowledge on the pathogenesis of neuroblastoma. In conclusion I recommend publication of the submitted manuscript without changes. Nevertheless, there are many interesting questions arising in regards to the manuscript, which the authors may wish to consider. Some of them are given below:

## Are there correlations between neuroblastoma patients concerning states of activation of their gene signaling pathways?

Author’s response: This is a very good question although including these results and commenting them is a bit away from the scope of this manuscript. Certainly, some circuits are correlated due to the dependence of some genes shared, which is an obvious correlation, but others not sharing genes are correlated as well, probably because they are under the same regulatory program. We have included a couple of sentences making reference to this comment at the end of the first paragraph of the Conclusions section.

## KM survival curves are quite asymmetric. Are there differences between survivals still seen if the group of patients is split into two equal size subgroups rather than in proportions 90% versus 10%?

Author’s response: The idea was to discover these circuits remarkable related to survival. Therefore we had to clearly distinguish patients with high mortality rate from those with a low mortality rate and we thus focused on the extremes of the distribution. Splitting into two groups would reduce the detection sensitivity by including many patients with an intermediate survival in both groups.

## Is it possible to relate pathogenic status of gene signaling pathways, discovered in the data, to somatic mutations in certain genes?

Author’s response: Probably, but there is no much information in TCGA regarding somatic mutations in neuroblastoma to reach solid conclussions.

## Is the aspect of multiple testing addressed in the computations?

Author’s response: Yes, actually FDR is used although was not explicitly stated in the text because we referred to the original publication. However, the referee is right in noting this absence and we have explained the correction used (FDR) in a new subsection within the Methods section.

## How one can image the computed status of gene signaling pathways in the context of cancer progression? Should one expect that the status of activation/inhibition changes during the evolution of cancer? Is it possible to observe some correlations with cancer pathogenic stages?

Author’s response: We are pretty sure that a time-series circuit activity study would reveal very interesting results. The only coarse grain approach to study time progression of circuit activities in cancer we did is in the original paper describing the method (Hidalgo et al., 2017) where we show how circuits corresponding to different cell functionalities changed across cancer stages. Some of them were initially activated in stage I and then remain with a similar activity, and we attributed them to cancer initiation functionalities, and some other increased its activity along cancer stages, and we guessed they were related to cancer progression cell functionalities.

## Additional files

Additional file 1: Schema of signaling circuit definition**.** A) Different types of circuits can be defined within the pathways as subpathways that connect receptor proteins (those that receive the signal) to effector proteins (those at the end of the pathways). and ultimately to the functions behavioral outcomes triggered by the effector proteins. B) Elementary signaling circuits are defined as subpathways that connect a unique receptor to a unique effector protein; C) Effector circuits are defined as subpathways that connect a unique effector protein to all possible receptor protein that can start the transduction of signal; D) functional circuits are defined as subpathways that connect all the possible receptor proteins that can transduce signal to all the possible effector proteins that trigger a unique function. E) the concept of functional circuits is very convenient to define subsets of domain-specific functional circuits of relevance for the problem studied. For example, in cancer, the well-known cancer hallmarks can be identified among the GO terms of the functions triggered by the effector proteins. (JPG 250 kb)

Additional file 2: Schema of the model of signaling circuit dynamics. A) The dynamics of the circuit is modeled taking the normalized gene expression values as proxies of protein activation status (the more expressed is a gene. The more likely the corresponding gene product will be active); B) given that both activations and repression activities can occur along the pathway, different scenarios are considered for the transmission of the signal: only activation, simultaneous activation and inhibition and only inhibition, which are coded in the formula; C) the application of the formula provided estimations of the different fluxes of signal across the circuit that finally arrive to the effector protein. In this way, a gene expression profile can be transformed in the corresponding signaling circuit activity profile (or functional profile). (JPG 149 kb)

## Abbreviations

FDR: False Discovery Rate
FPKM: Fragments Per Kilobase of transcript per Million
KEGG: Kyoto Encyclopedia of Genes and genomes
K-M: Kaplan-Meier curves
