# Supplementary figures and images for: Models of cell signaling uncover molecular mechanisms of high-risk neuroblastoma and predict disease outcome
Source: Biol Direct. 2018 Aug 22;13:16. doi: 10.1186/s13062-018-0219-4 (PMC6106876; doi:10.1186/s13062-018-0219-4)

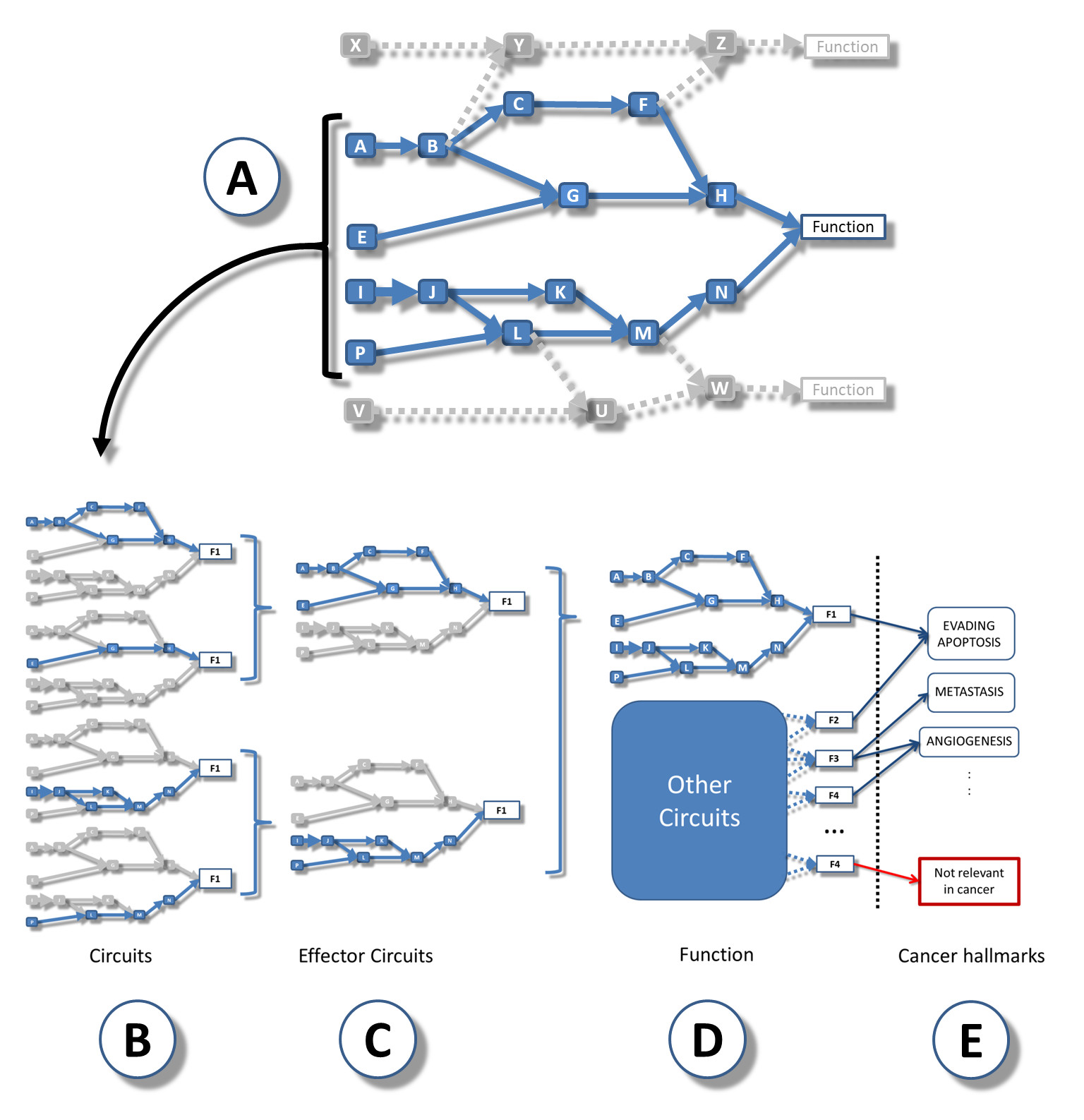

Supplement: Supplementary file 1 — Schema of signaling circuit definition. A) Different types of circuits can be defined within the pathways as subpathways that connect receptor proteins (those that receive the signal) to effector proteins (those at the end of the pathways). and ultimately to the functions behavioral outcomes triggered by the effector proteins. B) Elementary signaling circuits are defined as subpathways that connect a unique receptor to a unique effector protein; C) Effector circuits are defined as subpathways that connect a unique effector protein to all possible receptor protein that can start the transduction of signal; D) functional circuits are defined as subpathways that connect all the possible receptor proteins that can transduce signal to all the possible effector proteins that trigger a unique function. E) the concept of functional circuits is very convenient to define subsets of domain-specific functional circuits of relevance for the problem studied. For example, in cancer, the well-known cancer hallmarks can be identified among the GO terms of the functions triggered by the effector proteins. (JPG 250 kb) [file 13062_2018_219_MOESM1_ESM.jpg]

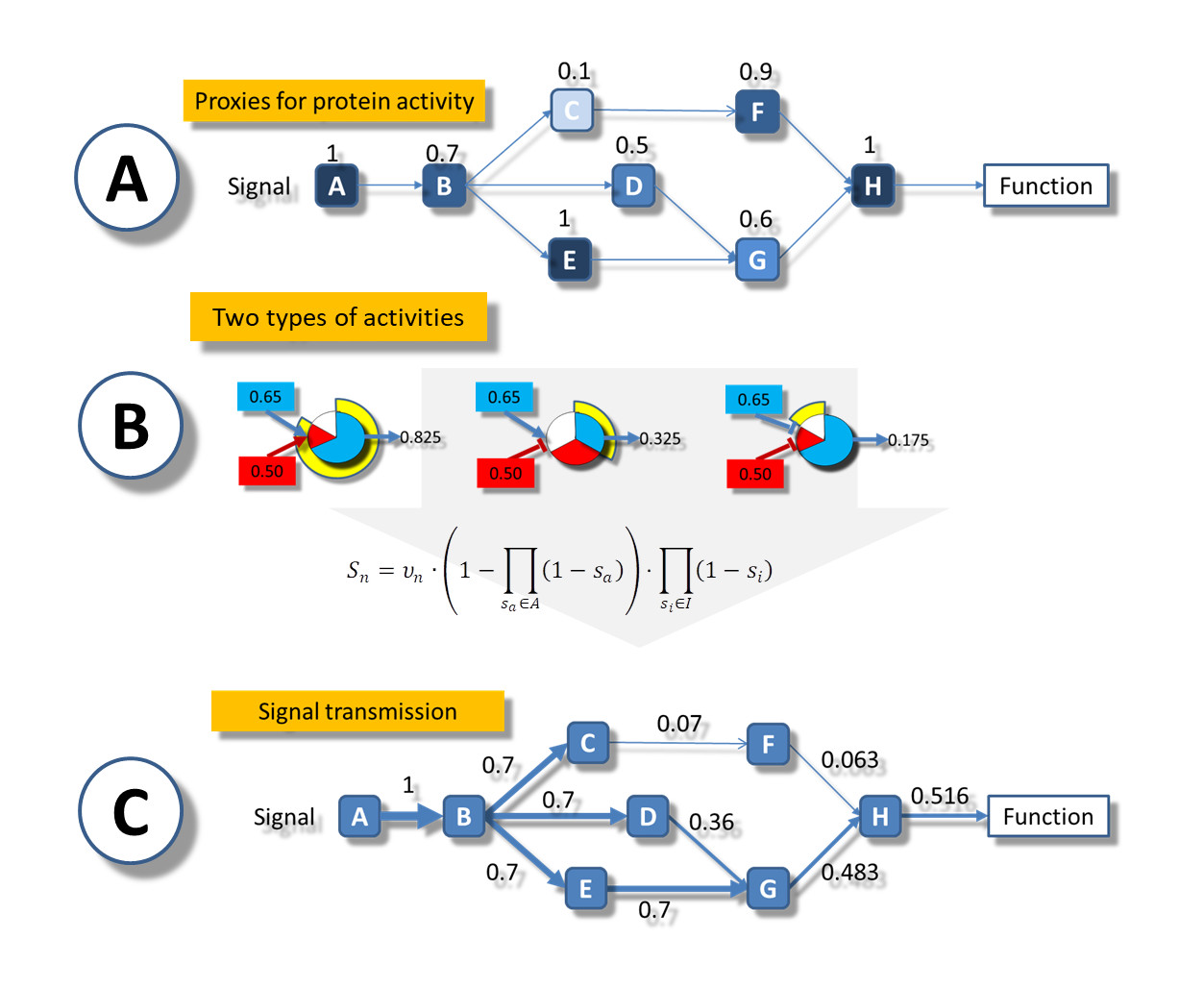

Supplement: Supplementary file 2 — Schema of the model of signaling circuit dynamics. A) The dynamics of the circuit is modeled taking the normalized gene expression values as proxies of protein activation status (the more expressed is a gene. The more likely the corresponding gene product will be active); B) given that both activations and repression activities can occur along the pathway, different scenarios are considered for the transmission of the signal: only activation, simultaneous activation and inhibition and only inhibition, which are coded in the formula; C) the application of the formula provided estimations of the different fluxes of signal across the circuit that finally arrive to the effector protein. In this way, a gene expression profile can be transformed in the corresponding signaling circuit activity profile (or functional profile). (JPG 149 kb) [file 13062_2018_219_MOESM2_ESM.jpg]
